# Quantification of Free Short-Chain Fatty Acids in Raw Cow Milk by Gas Chromatography-Mass Spectrometry

**DOI:** 10.3390/foods12071367

**Published:** 2023-03-23

**Authors:** Xufang Wu, Fengen Wang, Meiqing Chen, Jiaqi Wang, Yangdong Zhang

**Affiliations:** 1 Laboratory of Quality and Safety Risk Assessment for Dairy Products of Ministry of Agriculture and Rural Affairs, Institute of Animal Sciences, Chinese Academy of Agricultural Sciences, Beijing 100193, China; 2 Milk and Dairy Product Inspection Center of Ministry of Agriculture and Rural Affairs, Institute of Animal Sciences, Chinese Academy of Agricultural Sciences, Beijing 100193, China; 3 State Key Laboratory of Animal Nutrition, Institute of Animal Sciences, Chinese Academy of Agricultural Sciences, Beijing 100193, China; 4 Institute of Quality Standard and Testing Technology for Agro-Products, Shandong Academy of Agricultural Sciences, Jinan 250100, China

**Keywords:** milk, free short-chain fatty acids, gas chromatography-mass spectrometry

## Abstract

Free short-chain fatty acids (FSCFAs) are a momentous contributor to the flavor of the raw cow milk. Hence, the purpose of this research was to build an approach for the quantification of 10 FSCFAs in raw cow milk. Raw cow milk samples are acidified by hydrochloric acid ethanol (0.5%) solution pretreatment and then processed on the gas chromatography-mass spectrometry. With the exception of iso C5:0 and anteiso C5:0 co-flux, the remaining eight FSCFAs were effectively separated by chromatography. The methodological validation data revealed that the linear relationship satisfied the assay requirements (coefficient of determination >0.999), the limits of quantification were 0.167 to 1.250 μg mL^−1^, the recoveries ranged from 85.62% to 126.42%, the coefficients of variation were 1.40~12.15%, and no SCFAs in the triglyceride form were potential degradation, and the precision ranging from 0.56% to 9.09%. Our easy, fast, and robust method successfully determined three FSCFAs in raw cow milk without derivatization. Some characteristic features of FSCFAs have been discovered in raw cow milk such as its higher percentages of C4:0 and C6:0. Our research has provided a very valuable method for the future quality and safety control of raw milk and nutritional studies.

## 1. Introduction

Milk is named “white blood” and “liquid bread”, which has the double nutritional functions of “basic nutrition” and “active nutrition” [1,2]. Milk is not only a nutritionally rich food, with essential nutrients such as lactose, protein, fat, vitamins (vitamin A, vitamin E, vitamin C, and B vitamins) and minerals (calcium, iron, zinc, and potassium), but also has active nutrients such as lactoferrin, lactoglobulin and whey protein. Raw milk has a characteristic milky flavor [3,4], which makes it more attractive to consumers. Based on the literature, free fatty acids, especially free short-chain fatty acids (FSCFAs), are critical factors which affect the milk flavor [3,5], and increased FSCFAs are the responsible for the sourness of milk [6]. Previous research has described C2:0 as the vinegary, pungent sourness of raw milk produced during storage [7], which is also an important source of flavor in yoghurt products, and the nauseating, sweet, rancid cheese-like odor of C6:0 [8]. Raw milk spoilage can occur when bacteria and microbial metabolism in raw milk cause off-flavors and taste or texture changes that make raw milk unfit for human consumption and dairy processing. It has been found that there is a strong correlation between SCFAs produced by bacteria, microbial metabolism or bacterial lipase release in raw milk and the organoleptic acceptability of raw milk [9,10]. Therefore, milk changes in FSCFAs may reveal potential indicators of raw milk spoilage. Additionally, FSCFAs have exhibited positive effects in supplying energy sources [11,12], anti-oxidation [13], enhancing brain development [14], reducing hypertension and hyperlipidemia [15,16]. They have anti-allergy effects [17], preventing colonic inflammation [18] and cardiovascular disease [19], and prevent obesity [11,20]. Consequently, an accurate quantification of FSCFAs in milk is very valuable for the quality and safety control of raw milk and for nutritional studies.

FSCFAs are characterized by a small molecular mass, with a high polarity, volatility and water solubility. The quantitative analysis of FSCFAs has drawn the attention of an increasing number of scholars, and the determination of FSCFAs in milk [21] and cheese [21,22,23] has been investigated. Multiple analytical platforms have been utilized to milk FSCFAs’ quantitation, including liquid chromatography-mass spectrometry [24,25], gas chromatography (GC) coupled with flame ionization and GC coupled with mass spectrometry (GC-MS) [26,27,28]. GC-MS was the most commonly used platform for FSCFAs’ determination. Previous literature reports on the derivatization of FSCFAs in cow’s milk and cheese into a larger molecular weight, with higher non-polarity, less volatility and less water-soluble FSCFAs ester [21,26,29], which requires a much longer and more complicated sample pretreatment process. In addition, the loss of FSCFAs in derivatization is inevitable. Therefore, a novel headspace injection has been used for the detection of FSCFAs in milk for humans [28]. In recent years, the direct injection after acidification to determine FSCFAs has gradually entered researchers’ vision [27]. For the reason of the complexity of the milk matrix, especially that the proteins are likely to interfere with the determination and even affect the performance of the instrument, as much protein as possible must be removed from the milk. A part of the milk proteins (glycoproteins, etc.) is distributed on the milk lipid globule membrane [30], combined firmly with lipids, which are not easily soluble in water, dilute salt solution, and dilute acid or dilute alkali, so organic solvents like ethanol and butanol with certain hydrophilicity and strong esterophilicity are chosen as the ideal reagents for precipitating proteins in milk. In addition, since FSCFAs are extremely easily ionized in water, the pH is important for the determination of FSCFAs [27]. Therefore, in the previous reports, hydrochloric acid and metaphosphoric acid were often used to inhibit the ionization of human milk FSCFAs in water [27].

In general, although only a minor fraction of raw milk is FSCFAs, the condition of freshness or rancidity of raw milk is highly relevant to FSCFAs. Consequently, the purpose of this study was to establish a GC-MS method for a simultaneous and accurate determination of 10 FSCFAs in raw milk without derivatization by using hydrochloric acid ethanol to precipitate proteins in raw milk samples, providing a science-based approach for raw cow milk quality and safety control, and to further nutritional function studies.

## 2. Materials and Methods

### 2.1. Materials and Reagents

In 2022, 21 samples of raw cow’s milk were kindly supplied by the Institute of Animal Science, at the Chinese Academy of Agricultural Sciences. A whey powder solution was produced by a mixture of whey powder (1 g), which was purchased from the local market, and Ultrapure water (35 mL). All samples were stored at −40 °C.

FSCFAs standards including C1:0, C2:0, C3:0, iso C4:0, C4:0, iso C5:0, anteiso C5:0, C5:0, iso C6:0, C6:0, and internal standard (anteiso C6:0) were purchased from Dr. Ehrenstorfer GmbH (Augsburg, Germany). C2:0 TAG, C4:0 TAG, and C6:0 TAG were purchased from the Shanghai Yuanye Biotechnology Co., Ltd. (Shanghai, China).

Ethanol (MS grade) was purchased from Merck (Darmstadt, Germany), hydrochloric acid (purity ≥ 98%) was purchased from the Macklin Co., Ltd. (Shanghai, China). Ultrapure water was filtered by the Milli-Q purification system (Millipore, Bedford, MA, USA).

### 2.2. Sample Preparation

Our raw cow’s milk sample preparation was referred to by Jiang et al. [27] with modification. Briefly, the frozen raw cow’s milk samples of Holstein cows were thawed in a water bath at 37 °C, stirred to avoid foaming. Then, 1 mL of the raw cow’s milk sample was pipetted and 50 μL of internal standard solution (2500 μg mL^−1^) was added. Next, 3 mL of hydrochloric acid/ethanol (0.5%) solution and 1 mL of Utrapure water were added to the samples, vortexed and mixed, and centrifuged at 12,000× *g* for 20 min at 4 °C; 1 mL of the supernatant was pipetted into the injection vial, and then the GC-MS measurement was performed.

### 2.3. Standards Preparation

The FSCFAs standards were prepared following the same protocol as the raw cow’s milk samples. Briefly, 600 μL hydrochloric acid/ethanol (0.5%) solution was added to the FSCFAs standards and fixed to 1 mL with ultrapure water. The FSCFAs standards were diluted to 200, 100, 50, 20, 10, 5, 2, and 1 μg mL^−1^. The internal standard concentration of 25 μg mL^−1^ was used for quantification. All standard solutions were prepared and stored in ampoules at 4 °C.

### 2.4. GC-MS Analysis Procedure

The Agilent 6890A/6895C GC-MSD system (Agilent Technologies, Santa Clara, CA, USA) combined with a DB-FFAP capillary column (30 m × 250 μm × 0.25 μm, Agilent Technologies, CA, USA) were used for the analysis. Ultrahigh purity helium (99.999%) carrier gas flow was constant at 1 mL/min. One µL of FSCFA extraction liquid was injected via an autosampler into the GC inlet operated in a 20:1 spit mode. The inlet temperature was 250 °C and the overall operation time was 26 min. The oven temperature was set at an initial temperature of 50 °C (held for 1 min), and then increased at a rate of 10 °C min^−1^ to 170 °C (held for 2 min), and finally ramped up to 240 °C at a rate of 50 °C min^−1^ (held for 9.6 min).

The MS ion source, quadrupole and transfer line, were held at 230 °C, 150 °C, and 250 °C, respectively. The ionizing energy was set at 70 eV with a solvent delay of 4.5 min. The quantification of FSCFAs was carried out using the selected ion monitoring mode (Table 1). The individual identification of FSCFAs was based on comparing the retention time, 1 quantitative ion and 3 qualitative ions with the standard. To enhance sensitivity, the quantitative fragment ion of each FSCFA with the best signal-to-noise ratio was selected, and the run time was partitioned into 4 windows (including the internal standard), in which the dwell time was 10 ms and the scanning frequency was >12 cycle/s. The GC-MS parameters were showed in Table 1.

### 2.5. Methodological Validation

Our method validation was conducted in accordance with the International Conference on Harmonization guidelines (ICH Q2 (R1) Guide) [31]. Linearity ranged from 1–200 μg mL^−1^. Corresponding injection concentrations of signal-to-noise = 3; 10 were selected on the ion flow diagram, and the limit of detection (LOD) and the limit of quantification (LOQ) of each FSCFA were calculated. The recoveries and coefficients of the variation of FSCFAs were calculated by adding 10 FSCFAs to the whey solution in 3 concentration gradients of 10, 50 and 200 μg mL^−1^, respectively. Moreover, the potential degradation of triglycerides was also calculated with the same procedure of FSCFAs recoveries. On this basis, precision data (Intra and inter day) were calculated for three consecutive injections and three days within 24 h at three concentration gradients of 10, 50 and 200 μg mL^−1^, respectively.

### 2.6. Statistical Analysis

The peak area values and chromatograms of the FSCFAs were obtained by manual integration with the Agilent MSD Chemistry Integrator E.02.02.1431. Statistical, and the analysis of data results were performed by Excel 2021.

## 3. Results and Discussion

### 3.1. Optimization of Chromatographic Conditions

**Figure 1:**
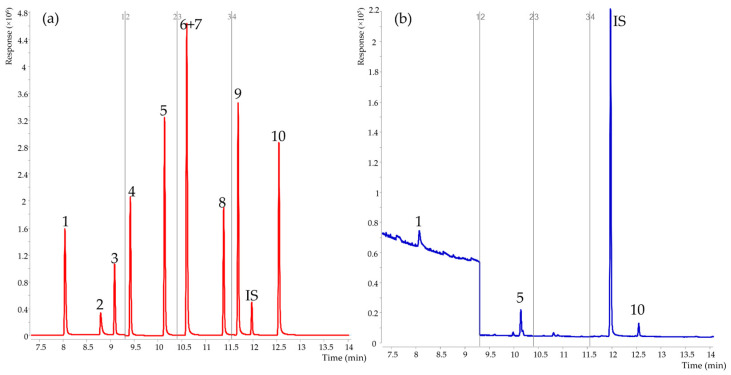
Total ion chromatogram of 10 FSCFAs by GC-MS. (**a**) Red line, total ion chromatogram of standards in water (**b**) Blue line, total ion chromatogram of raw cow’s milk. 1, C2:0; 2, C1:0; 3, C3:0; 4, iso C4:0; 5, C4:0; 6, iso C5:0; 7, anteiso C5:0; 8, C5:0; 9, iso C6:0; 10, C6:0; IS, anteiso C6:0.

FSCFAs are characterized by strong polarity and volatility, so a strongly polar nitro-p-phenylene terephthalic acid-modified polyethylene glycol-filled DB-FFAP capillary column designed for the analysis of volatile fatty acids was selected for quantification. As shown in Figure 1, 10 FSCFAs were detected simultaneously by this approach, with the exception of iso C5:0 and anteiso C5:0 co-flux, which were able to achieve an effective chromatographic separation of the remaining 8 FSCFAs. By comparative analysis of the retention time and the mass spectrometric fragment ions of iso C5:0 and anteiso C5:0 standards, it was noticed that both iso C5:0 and anteiso C5:0 were consistent in retention time and similar in mass spectrometric fragment ions, which could not be separated effectively by characteristic ions. In prior research, the large majority of the literature has effectively quantified only anteiso C5:0 and C5:0 in biological samples [27,32,33]. Coelution of iso C5:0 and anteiso C5:0 after acidification has been reported in other literature [34], and in that research the HP-inno Wax column with the stationary phase of nitro-p-phenylene terephthalic acid-modified polyethylene glycol was also used for chromatographic separation and mass spectrometric quantification, where the problem of co-eluting peaks was remarkably consistent with the results of this study. According to the literature, branched-chain FSCFAs in cow’s milk mainly originate from the production of protein or amino acid fermentation valine, leucine, and isoleucine when the energy supply of the dairy organism exceeds the demand [12,28], and the content of branched-chain FSCFAs in cow’s milk is extremely low. Therefore, the proposed method in this experiment can meet the need for the detection of FSCFAs in cow’s milk.

Furthermore, in order to better improve the response values and the sensitivity of the targets, the separation effects of the 10 FSCFAs standards were compared in this study with a split ratio of 10:1 and 20:1, a carrier gas flow rate of 0.5 mL min^−1^, 1 mL min^−1^, 1.5 mL min^−1^ and different programmed warming modes. The results showed that the best separation of the 10 FSCFAs was achieved with a split ratio of 20:1, a carrier gas flow rate of 1 mL min^−1^ and the warming conditions described in Section 2.3.

### 3.2. Methodological Validation

As shown in Table 2, regression equations fitted to the standard curves of FSCFAs were broadly applicable within the range of 1200 μg mL^−1^, with coefficients of determination above 0.999. The instrument limits of detection and quantification of FSCFAs were 0.064–0.375 μg mL^−1^ and 0.167–1.250 μg mL^−1^, respectively. Assuming that the total fatty acid content of the raw cow’s milk lipid was 4%, the minimal relative content of FSCFAs in raw cow milk would be 0.0004–0.0031 g/100 g of fatty acids. The recoveries and precision were determined by adding different concentrations of FSCFAs and SCFAs-triglyceride standards to the blank whey solution with 4 replicates for each spiked level, and the pretreatment method was the same as that for raw cow’s milk samples. As listed in Table 3, intra-day and inter-day precision of this assay ranged from 0.92–8.68%, and 0.56–9.09%, with recoveries of 85.62–126.42% and coefficients of variation 1.40–12.15% for FSCFAs. There was no potential degradation on the bound SCFAs in raw cow’s milk in the form of triglycerides.

### 3.3. Content of FSCFAs in Raw Cow Milk

As shown in Table 4, the C2:0, C4:0 and C6:0 FSCFAs were detected in raw cow’s milk, and the rest were not detected. The total amount of FSCFAs in raw cow’s milk was 14.63 μg mL^−1^ and the proportions of free C4:0, C6:0 and C2:0 in raw cow’s milk were 43.77%, 32.24% and 23.99%, respectively. The high level of free C4:0 in raw cow’s milk originates from β-hydroxybutyric acid produced by the fermentation of carbohydrates by rumen microorganisms, which is transported through the bloodstream to the mammary gland to be reduced to free C4:0 [35]. In addition, we found little detectable presence of branched-chain FSCFAs in raw cow’s milk, which may be related to the fact that branched-chain FSCFAs occur mainly in cows with low energy supply, where rumen microorganisms turn to fermentation of feed proteins and amino acids, for example, to produce iso C4:0, iso C5:0, anteiso C5:0, and iso C6:0, from the branched-chain amino acids valine, isoleucine and leucine [12].

### 3.4. Comparison of Previous GC-MS Methods with the Method Reported Here

GC-MS have great advantages to the capacity for fatty acid detection in milk [36]. Table 5 shows a brief comparison of the GC-MS technique for the detection of FSCFA in raw milk compared to the current study. Previous research using derivatization techniques to determine FSCFAs in cow milk [21,26]. However, the derivatization process requires laborious sample preparation and longer pretreatment times, with the potential loss of free short-chain fatty acids, resulting in a reduced sensitive of the assay and a reduction in the detected number of FSCFAs. Headspace injection has also been used for the direct determination of FSCFAs in human milk [28]. The headspace injection eliminates the need for lengthy sample pretreatment and avoids the introduction of organic solvents to interfere with the analysis. On the other hand, achieving gas-liquid equilibrium in the headspace injection process is prone to errors, resulting in poor sample parallelism. Previous studies have also used relative response factors to calculate FSCFAs in raw cow milk samples, which is carried out by dividing the ratio of the peak area of each FSCFAs to the internal standard by the mass ratio of the two to obtain its content in raw cow milk [37]. One more step, Jiang et al. [27] has developed a semi-quantitative method for the determination of FSCFAs in human milk based on the concentration and peak area of the internal standard 2-ethylbutyric acid and the peak area of the target. Our method is a further optimization of the Jiang et al. method [27]. As shown in Table 5, compared with the Jiang et al. [27] research, the current method in our study was able to detect more species of FSCFAs and was more accurate by the internal standards for quantification. However, we detected fewer FSCFAs in the actual milk sample compared to Jiang et al. [27]. Therefore, the following reasons have be speculated. Firstly, FSCFAs are less abundant in raw milk and the quantification limit of the instrument was to ensure that the target peaks without solvent interference, so fewer species of FSCFAs were detected. Secondly, compared to cows, humans consume less dietary fiber-based substances and more refined carbohydrates and proteins from their diet, and human milk contains more types of FSCFAs than cow milk due to the different substrates fermented by the human and cow organisms.

## 4. Conclusions

In this study, a simple, rapid GC-MS method without derivatization was developed for the simultaneous quantitative analysis of 10 FSCFAs in cow milk. The methodological validation data showed that the method had good linearity, the limits of detection and quantification could meet the detection of FSCFAs in milk, the accuracies were high, and the precision was good. Therefore, the present study method can be used for the quantitative determination of FSCFAs in milk, providing a scientific basis for milk quality and safety control and the further evaluation of the nutritional value and physiological functions of FSCFAs in milk. Compared with previous studies, the present method focuses on other isomers of FSCFAs with less content in milk, and there is some progress in the detection throughput of the targets, but there is also a defect in that iso C5:0 and anteiso C5:0 co-elution cannot be separated, and further optimization of the instrument conditions is needed to achieve the accurate quantification of the 10 FSCFAs. In general, our research provided a scientific method for milk FSCRAs detection and the future must show if the method has the potential as a tool for evaluation freshness of raw milk.

## Figures and Tables

**Table 1:** GC-MS parameters for determination of free short-chain fatty acids (FSCFAs).

Compounds	Window No.	Start Time (Min)	Retention Time (Min)	Quantitative Ion (m/z)	Qualitative Ion (m/z)			Dwell Time (ms)
C2:0	1	4.5	8.077	43	60	28	45	10
C1:0			8.900	46	43	28	29	
C3:0			9.119	74	28	45	57	
iso C4:0	2	9.3	9.452	43	73	88	60	10
C4:0			10.172	60	73	43	88	
iso C5:0/anteiso C5:0	3	10.5	10.641	43	87	60	74	10
C5:0			11.416	60	43	55	74	
iso C6:0	4	11.6	11.720	74	87	43	55	10
C6:0			12.579	60	43	73	55	
IS ^1^			12.006	60	43	87	55	

^1^ IS, internal standard (anteiso C6:0).

**Table 2:** Linearity and sensitivity of the proposed method.

Compounds	Regression Equation	Linearity Range (μg mL^−1^)	R^2 1^	LOD (μg mL^−1^) ^1^	LOQ (μg mL^−1^) ^1^
C2:0	y = 0.4641x + 0.0292	1~200	0.9992	0.375	1.250
C1:0	y = 0.2505x + 0.0041	1~200	0.9998	0.068	0.227
C3:0	y = 0.5629x + 0.0265	1~200	0.9990	0.050	0.167
iso C4:0	y = 0.8367x + 0.0358	1~200	0.9992	0.100	0.333
C4:0	y = 1.7464x + 0.0627	1~200	0.9994	0.064	0.213
iso C5:0/anteiso C5:0	y = 0.3631x + 0.0225	1~200	0.9990	0.140	0.465
C5:0	y = 1.2557x + 0.0364	1~200	0.9996	0.107	0.357
iso C6:0	y = 1.6426x + 0.0808	1~200	0.9995	0.167	0.556
C6:0	y = 1.1568x + 0.0337	1~200	0.9997	0.171	0.571

^1^ R^2^, coefficient of determination; LOD, the limits of detection; LOQ, the limits of quantification.

**Table 3:** Recovery experiments and precision of 10 FSCFAs and the potential degradation of 3 SCFAs triglycerides spiked in whey solution (n = 4).

Compounds	Spiked Level											
	10 µg mL^−1^				50 µg mL^−1^				200 µg mL^−1^			
	R ^1^	% CV	% RSD 1 ^1^	%RSD 2 ^1^	R	% CV	% RSD 1	%RSD2	R	% CV	% RSD 1	% RSD2
C2:0	116.26	6.35	3.17	1.52	103.01	1.40	1.21	1.66	98.81	2.32	7.23	0.99
C1:0	126.42	3.61	7.94	8.83	89.91	10.40	3.47	8.04	104.21	2.81	8.68	2.63
C3:0	86.75	3.53	1.58	1.29	91.76	2.45	1.32	0.56	89.38	12.15	2.19	3.78
iso C4:0	86.35	3.94	1.29	2.85	96.67	2.18	1.56	0.85	97.07	2.29	2.27	1.07
C4:0	88.10	2.75	1.68	2.19	94.04	2.07	1.02	0.95	97.14	1.73	1.73	0.71
iso C5:0/anteiso C5:0	85.62	5.43	1.73	6.79	102.35	2.23	1.45	1.32	98.99	1.92	3.93	1.22
C5:0	88.97	3.09	1.42	3.36	94.94	1.41	1.01	1.34	98.86	1.51	2.92	3.15
iso C6:0	86.46	9.64	1.50	9.09	97.02	2.62	1.49	2.58	99.95	2.72	5.22	1.97
C6:0	88.11	3.97	0.92	3.78	95.86	1.77	1.64	1.71	99.54	1.98	3.69	1.21
C2:0 TAG ^2^	ND	ND	/	/	ND	ND	/	/	ND	ND	/	/
C4:0 TAG	ND	ND	/	/	ND	ND	/	/	ND	ND	/	/
C6:0 TAG	ND	ND	/	/	ND	ND	/	/	ND	ND	/	/

^1^ R, Recoveries; % RSD 1, Intra-day % RSD; % RSD 2, Inter-day % RSD. ^2^ TAG, triglycerides.

**Table 4:** Content of FSCFAs in raw cow’s milk (n = 21).

Compounds	Content (µg mL^−1^)	% of Total FSCFAs
C2:0	3.51	23.99
C1:0	ND ^1^	0.00
C3:0	ND	0.00
iso C4:0	ND	0.00
C4:0	6.40	43.77
iso C5:0/anteiso C5:0	ND	0.00
C5:0	ND	0.00
iso C6:0	ND	0.00
C6:0	4.72	32.24

^1^ ND, not detection.

**Table 5:** A comparison of previous methods in raw milk with the method reported here.

Sample	Sample Preparation				Instrument Method			Quantification	Samples FSCFAs Species	Reference
	Derivatization	IS	Reagents	Preparation Time	Run Time	FSCFAs Number	LOQ (μg mL^−1^)			
cow milk	yes	\	Boron trifluoride butanol	long	34.67 min	2	20	external quantification	C4:0, C6:0	[21]
human milk	no	2-ethylbutyric acid	hydrochloric acid/ethanol	short	14 min	6	\	semi-quantitative	C1:0, C2:0, C3:0, iso C4:0, C4:0, C6:0	[27]
human milk	no	\	\	short	15 min	1	\	external quantification	C4:0	[28]
cow milk	yes	Isotope	ethyl chloroformate, ethanol, pyridine	long	50 min	2	0.067	IS quantitative	C4:0, C6:0	[26]
cow milk	no	1,3-butanediol	\	short	28.5 min	7	\	relative response factor	C2:0, C3:0, iso C5:0, C6:0	[37]
cow milk	no	anteiso C6:0	hydrochloric acid/ethanol	short	26 min	10	0.167–1.250	IS quantitative	C2:0, C4:0, C6:0	current

## Data Availability

The data presented in this study are available on request from the corresponding author.
